# Pixel-Wise Statistical Mapping with Voltage-Sensitive Dye Imaging Reveals Differential Hippocampal Circuit Effects of Bisphenol A and Related Phenolic Analogs

**DOI:** 10.1523/ENEURO.0165-26.2026

**Published:** 2026-10-09

**Authors:** Yoko Tominaga, Makiko Taketoshi, Kentaro Tanemura, Takashi Tominaga

**Affiliations:** ^1^Institute of Neuroscience, Tokushima Bunri University, Takamatsu, Kagawa 760-8542, Japan; ^2^Optical Neuroimaging Unit, Okinawa Institute of Science and Technology Graduate University, Onna, Okinawa 904-0495, Japan; ^3^Laboratory of Animal Reproduction and Development, Graduate School of Agricultural Science, Tohoku University, Sendai, Miyagi 981-8555, Japan; ^4^Kagawa School of Pharmaceutical Sciences, Tokushima Bunri University, Takamatsu, Kagawa 760-8542, Japan

**Keywords:** BPA, environmental chemical, hippocampus, optical recording, voltage-sensitive dye

## Abstract

Exposure to environmental chemicals such as bisphenol A (BPA) may affect neural circuit function in subtle and spatially distributed ways that are difficult to attribute to a single circuit element. Here, we developed a voltage-sensitive dye (VSD) imaging analysis pipeline for acute male mouse hippocampal slices. This approach measures stimulus-evoked fractional changes in VSD fluorescence $\left(\Delta F/F_{0}\right)$ to generate pixel-wise normalized maps, averages response magnitude within anatomically defined regions of interest (ROIs), and visualizes control-versus-compound differences using pixel-wise statistical maps before and after false discovery rate (FDR) adjustment. We applied this pipeline to compare the acute effects of BPA with those of two structurally related phenolic analogs. Evoked responses were quantified by their peak amplitude and time integral across hippocampal trisynaptic pathways at two stimulation levels determined from input–output relationships, with or without the $\mathrm{GAB}A_{A}$ receptor antagonist gabazine (SR-95531; 10 µM). The phenolic analogs reduced circuit responses across multiple ROI-defined conditions, whereas BPA produced comparatively limited effects. These alterations varied with pathway, subfield, age, response metric, and inhibitory state. These findings demonstrate the utility of integrating ROI averages with pixel-wise statistical maps for detecting condition-dependent circuit-level differences between structurally related compounds and indicate that BPA-related phenolic analogs can differ from BPA itself in their acute effects on hippocampal circuitry.

## Significance Statement

Exposure to environmental chemicals may alter brain circuit activity in subtle, spatially distributed ways. We developed a voltage-sensitive dye imaging pipeline for acute mouse hippocampal slices that combines regional summaries with pixel-wise statistical maps, quantifying regional response magnitude while localizing where statistically supported differences are expressed within evoked response fields. Using this framework, we compared the acute hippocampal circuit effects of bisphenol A (BPA) with those of two structurally related phenolic analogs. Under acute slice conditions, statistically supported response reductions were detected more consistently for the phenolic analogs than for BPA, depending on age, inhibitory state, hippocampal subfield, and response metric. These results show how integrated regional and pixel-wise analysis improves circuit-level phenotyping of structurally related compounds.

## Introduction

Environmental chemicals are widely encountered and difficult to avoid, and exposure may influence neural development and function; the central nervous system is a plausible target for such perturbations (Mendola et al., 2002; Grandjean and Landrigan, 2006, 2014; Bellinger, 2018; Aggarwal et al., 2022). However, linking chemical exposure to circuit-level physiological mechanisms remains challenging because functional effects may be subtle, spatially distributed, and dependent on developmental stage and network state.

Bisphenol A [BPA; 2,2-bis(4-hydroxyphenyl)propane] is widely used in polycarbonate plastics and epoxy resins and is commonly used as an experimental reference compound in studies of endocrine-disrupting chemicals (Rochester, 2013; Bao et al., 2020; La Merrill et al., 2020). BPA has been reported to affect endocrine signaling and multiple physiological systems, including neural and behavioral endpoints (Fisher et al., 1999; Wetherill et al., 2007; Palanza et al., 2008; Romano et al., 2016; Donato et al., 2017; Ma et al., 2019; Sasaki et al., 2023; Wang et al., 2023). At the same time, the increasing use of BPA-related phenolic compounds has raised concern about possible regrettable substitution and about whether structurally related compounds have similar biological activities (Trasande, 2017; Pelch et al., 2019). Whether such compounds produce similar or distinct acute effects on neural circuit function remains poorly understood.

Two such compounds, 4,4′-butylidenebis(6-tert-butyl-*m*-cresol) (BB; CAS 85-60-9) and 2,2′-methylenebis(6-tert-butyl-*p*-cresol) (MB; CAS 119-47-1), are structurally related phenolic antioxidants whose acute effects on hippocampal circuit function have not been directly compared with those of BPA under standardized physiological conditions.

The hippocampus provides a well-characterized and tractable circuit for quantifying mesoscale network dynamics because its trisynaptic pathways connecting the dentate gyrus (DG), CA3, and CA1 support robust stimulus-evoked propagation and are described by extensive theoretical and experimental frameworks (Eichenbaum, 2004; Squire, 2009; Bartsch and Wulff, 2015; Schapiro et al., 2017). Acute hippocampal slices offer a controlled preparation for comparative circuit-level phenotyping of perturbations that alter synaptic and network functions.

A key obstacle in circuit phenotyping is that region-of-interest (ROI) averaging provides a robust summary of regional response magnitude but does not resolve where within an evoked response field condition-dependent effects are spatially expressed. Single-site electrophysiology provides high temporal resolution but samples only restricted locations. Voltage-sensitive dye (VSD) imaging enables simultaneous measurement of fast membrane potential dynamics over large fields of view (Salzberg et al., 1973; Cohen et al., 1978; Grinvald and Hildesheim, 2004; Knöpfel and Song, 2019) and has been applied to quantify spatially extended activity patterns in hippocampal and cortical preparations (Tominaga et al., 2018; Kajiwara et al., 2019; Gusain et al., 2023; Milicevic et al., 2023; Utsumi et al., 2023; Ojima et al., 2024; Tsukuda et al., 2025). However, pixel-wise statistical evaluation of VSD imaging data remains less standardized than ROI-based analysis, particularly when spatial maps are interpreted together with slice-level replication and correction for multiple pixel-wise comparisons.

A framework that integrates ROI averages, uncorrected pixel-wise *p* value maps, and false discovery rate (FDR)-adjusted *q* value maps (Benjamini and Hochberg, 1995), while maintaining the hippocampal slice as the unit of replication (*n*), can help separate regional response magnitude, nominal spatial organization, and spatial effects that remain detectable after correction across pixels. In this framework, *p* value maps provide technically informative summaries of nominal spatial organization in VSD responses, whereas FDR-adjusted *q* value maps localize pixel-wise differences that remain detectable after correction for multiple comparisons.

Here, we present such an analysis framework and demonstrate its utility in a comparative chemical perturbation assay. The framework combines ROI averages, uncorrected pixel-wise *p* value maps (*p*-maps), and Benjamini–Hochberg FDR (BH-FDR)-adjusted *q* value maps (*q*-maps; Benjamini and Hochberg, 1995), with the hippocampal slice retained as the unit of replication throughout. Using this framework, we compared the acute hippocampal circuit effects of BPA with those of BB and MB across pathway-specific stimulation of the trisynaptic circuit, stimulation level, developmental stage, response metric, and inhibitory state (Juliandi et al., 2015; Chen et al., 2017; Hu et al., 2017; Bi et al., 2021; Sasaki et al., 2023; Xu et al., 2025). This study provides a practical analytical pipeline for spatially resolved comparative circuit phenotyping and a demonstration dataset illustrating how ROI averages, *p*-maps, and *q*-maps capture complementary levels of regional and spatial information.

## Materials and Methods

Unless otherwise noted, slice preparation, VSD staining, and optical recording procedures followed those described previously (Tominaga et al., 2000, 2019).

### Animals

C57BL/6N male mice aged 2 and 8 weeks (juvenile and young-adult stages, respectively) were used in accordance with protocols approved by the institutional Animal Care and Use Committee (approval numbers: KP20-83-2 and subsequent annual renewals through KP25-83-2). All applicable international, national, and institutional guidelines for the care and use of animals were followed. All experiments were performed between 2020 and 2025.

### Slice preparation

Transverse hippocampal slices (350 µm) were prepared in ice-cold artificial cerebrospinal fluid (ACSF) containing (in mM) 124 NaCl, 2.5 KCl, 2 CaCl_2_, 2 MgSO_4_, 1.25 NaH_2_PO_4_, 26 NaHCO_3_, and 10 glucose, equilibrated with 95% O_2_/5% CO_2_. Slices were placed on a fine-mesh membrane filter (Omnipore, JHWP01300, 0.45 µm pores) within a plexiglass ring and maintained in a moist, oxygenated chamber, as described previously (Tominaga et al., 2000; 2019). Slices were incubated at 28°C for 25 min before equilibrating to room temperature.

For each experimental day, slices were prepared from a single animal. Each slice was assigned to a single chemical condition (control, BPA, BB, or MB; see below, Chemical agents, for preparation details), such that chemical conditions were distributed across slices within animal/day when feasible. Slices were excluded for a given condition when recordings did not meet predefined quality requirements (e.g., unstable baseline or loss of viability) or when the stimulation/recording sequence for that condition could not be completed. Analyses were performed on an available-case basis, and *n* is reported for each condition and comparison.

### VSD staining

VSD staining was initiated after a 40 min stabilization period. Slices were stained for 20 min with 110 µl of VSD solution containing 0.2 mM Di-4-ANEPPS (dissolved in a mixture of ethanol, Cremophor EL, distilled water, fetal bovine serum, and ACSF) as described previously (Tominaga et al., 2000, 2019). For recording, slices were transferred to an immersion-style chamber and continuously perfused with oxygenated ACSF (1 ml/min) heated to 31°C (Tominaga et al., 2000).

### VSD imaging and optical recording

Imaging was performed using a tandem-lens epifluorescence macroscope configuration (Ratzlaff and Grinvald, 1991; Tominaga et al., 2000). The system utilized an f=20mm water-immersion objective lens (NA 0.35; Brainvision) and a PLAN APO 1.0× lens (No. 10446230; Leica Microsystems) as the projection lens. Illumination was provided by a stabilized green LED (LEX-2G; Brainvision) with appropriate filter sets (dichroic mirror, 575 nm; excitation, 530 nm; emission, 590 nm).

Because wide-field epifluorescence does not provide optical sectioning, the recorded VSD signal represents a depth-weighted population response rather than activity from a defined axial plane.

Fluorescence was recorded with a CMOS camera (MiCAM-02; Brainvision) at a sampling rate of 1.0 ms/frame. Each acquired image comprised 92×80 pixels. The calibrated sampling dimensions at the specimen plane were 15.625 µm/pixel in the x direction and 16.13 µm/pixel in the y direction, corresponding to a field of view of ∼1.44 × 1.29 mm. These values describe the spatial sampling of the recorded image; the optical point-spread function was not measured independently. Image processing, $\Delta F/F_{0}$ conversion, temporal and spatial filtering, and metric definitions are described in Preprocessing and metric definition.

### Experimental procedure

Stimulation was controlled via an Arduino Uno and ESTM-8 stimulator (Brainvision) using custom Igor Pro macros. To ensure slice stability, field excitatory postsynaptic potentials (fEPSPs) were monitored in the distal stratum radiatum of CA1. Stimulation was repeated every 30 s.

Stimulation intensities were set based on an input–output (stimulation–response) curve: low (L) was ∼20% of the maximal response (L: 10 V, 0.5 ms), and high (H) was near the plateau (near-maximal) response level (H: 40 V, 0.5 ms). For each condition, four recordings were averaged to produce one $\Delta F/F_{0}$ image sequence (time series) per slice per condition. Glass stimulating electrodes filled with ACSF (<1 MΩ) were placed at three locations targeting distinct segments of the hippocampal trisynaptic circuit (Fig. 1*A*): the Schaffer collateral pathway at the CA1/CA2 border region (stratum radiatum, ∼200 µm from the pyramidal cell layer) to evoke CA1 responses, the dentate hilar region near the opening of the DG to activate the mossy fiber pathway and evoke CA3 responses, and the molecular layer near the apex of the DG to stimulate the perforant path and evoke DG responses. These stimulation sites correspond to the CA1, CA3, and DG response fields, respectively, as referenced throughout the Results. Following baseline imaging, slices were incubated with gabazine (SR-95531; 10 µM) for at least 20 min before repeating the stimulation paradigm under identical conditions.

**Figure 1.**
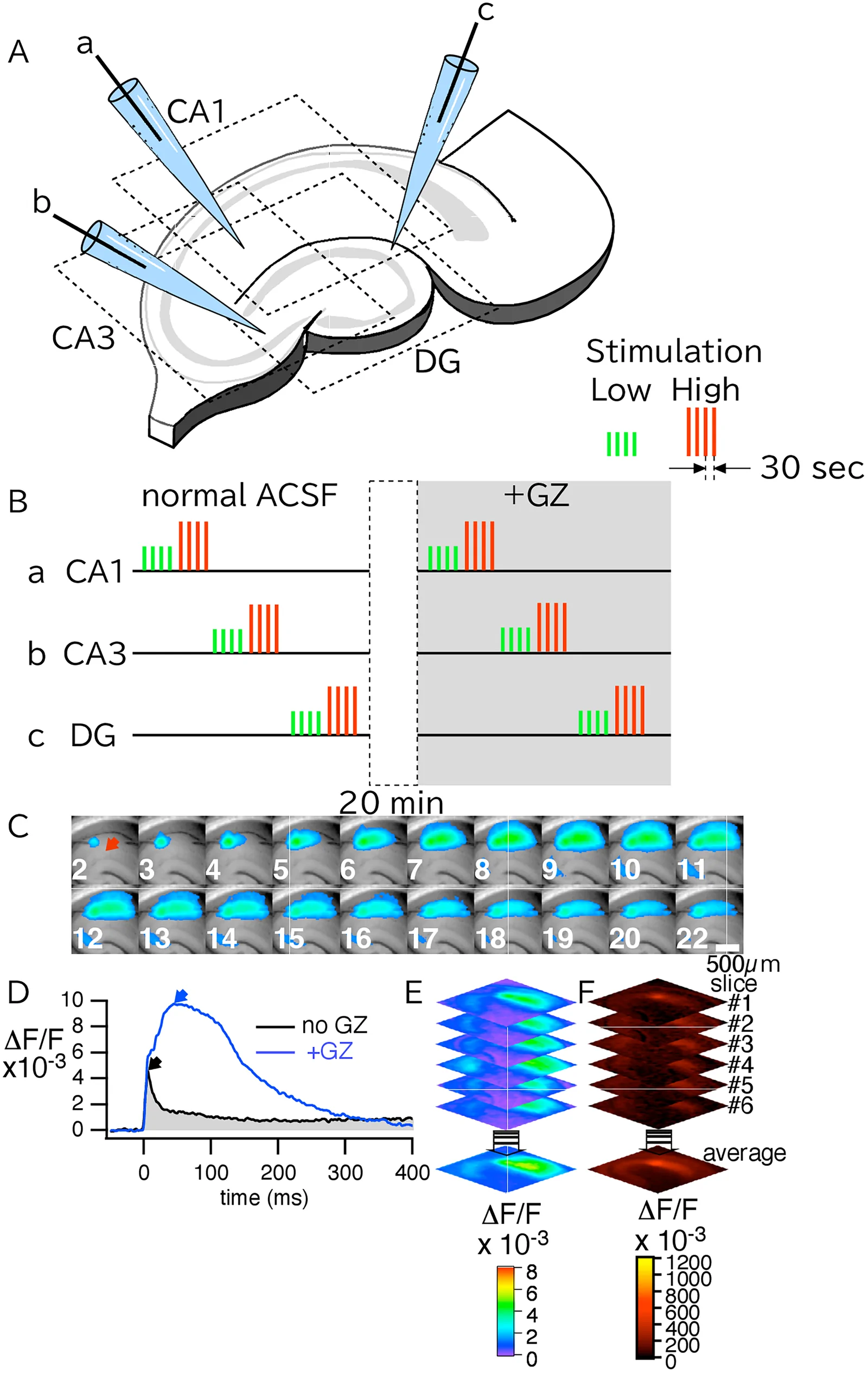
Experimental workflow for pathway-specific VSD imaging and pixel-wise map construction. ***A***, Schematic illustration of the hippocampal slice preparation and stimulation sites. ACSF-filled glass stimulating electrodes were placed at (a) the Schaffer collateral pathway at the CA1/CA2 border region (stratum radiatum, ∼200 µm from the pyramidal cell layer) to evoke CA1 responses, (b) the dentate hilar region near the opening of the DG to activate the mossy fiber pathway and evoke CA3 responses, and (c) the molecular layer near the apex of the DG to stimulate the perforant path and evoke DG responses. Dotted squares indicate the corresponding fields of view for VSD imaging in each region. ***B***, Stimulation and recording timeline. Under normal ACSF, four low-intensity stimulations (green) followed by four high-intensity stimulations (red) were applied separately at the CA1, CA3, and DG stimulation sites at 30 s intervals. After 20 min of perfusion with gabazine (+GZ; 10 µM), the same stimulation paradigm was repeated. ***C***, Representative time series of VSD snapshots (pseudocolored $\Delta F/F_{0}$) illustrating the spatiotemporal evolution of evoked activity following stimulation. Scale bar, 500 µm; the same spatial scale applies to all snapshots in ***C***. ***D***, Representative CA1 $\Delta F/F_{0}$ traces from an 8-week-old control preparation under normal ACSF (black; no GZ) and gabazine (blue; +GZ). Each arrowhead marks the PEAK of its trace. Gray shading shows SUM for the no-GZ trace over 0–400 ms; the same window was applied to both traces. ***E, F***, Pixel-wise map construction. For each slice, PEAK maps were computed as the maximum $\Delta F/F_{0}$ within the fixed poststimulus window (0–400 ms; ***E***), and SUM maps were computed as the time integral of $\Delta F/F_{0}$ over the same window (0–400 ms; ***F***). Slice-averaged maps were obtained by averaging the corresponding pixel-wise metric maps across slices (n=4−6).

Within each slice, recordings were obtained first under normal ACSF and then after gabazine application, and both low- and high-stimulation blocks were acquired using the same stimulation paradigm.

### Chemical agents

BPA (CAS 80-05-7; FUJIFILM Wako Pure Chemical), BB (CAS 85-60-9; Tokyo Chemical Industry), and MB (CAS 119-47-1; Tokyo Chemical Industry) were prepared as 50 mg/ml ethanol stocks. Final bath concentrations were 5 mg/L, equivalent to ∼21.9 µM (BPA), 13.1 µM (BB), and 14.7 µM (MB). Vehicle controls contained a matching ethanol concentration. This concentration was selected to allow direct mass-based comparison across compounds of differing molecular weights, consistent with previous in vivo studies of phenolic compounds (Sasaki et al., 2023).

### Statistical analysis

#### Definition of biological replicates (*n*)

In all analyses, the unit of observation (*n*) was the number of hippocampal slices. For each slice and condition, four trials were averaged to produce a single representative $\Delta F/F_{0}$ image sequence and derived maps. Pixels within an individual slice were not considered independent replicates to avoid sacrificial pseudoreplication (Hurlbert, 1984). Because one animal was used per experimental day and treatment conditions were distributed across slices from the same animal/day when feasible, animal/day was included as a blocking factor in the ROI analysis.

No a priori power calculation was performed. Sample size was determined by the complete set of available experiments that met the prespecified quality requirements and by the practical feasibility of the slice-recording design.

#### Preprocessing and metric definition

Fluorescence image sequences were converted to $\Delta F/F_{0}$, where $F_{0}$ was defined as the mean fluorescence over a fixed prestimulus baseline window from −45 to −2 ms relative to stimulus onset. For each slice and condition, four trials were averaged to obtain one $\Delta F/F_{0}$ image sequence. The image sequence was spatially and temporally filtered using a 5×5×3 Gaussian kernel, applied identically across all conditions.

From the slice-averaged $\Delta F/F_{0}$ image sequence, pixel-wise response metrics were computed over a fixed poststimulus window (0–400 ms; Fig. 1*D*). Peak-response amplitude (PEAK) was defined as the maximum $\Delta F/F_{0}$ value within this window (the arrowheads mark the respective PEAK values of the normal ACSF and gabazine traces in Fig. 1*D*), and response integral (SUM) was defined as the time integral (area under the response curve) of $\Delta F/F_{0}$ over the same window (shaded region in Fig. 1*D*; units of $\Delta F/F_{0}\cdot \mathrm{ms}$).

#### Pixel-wise statistics and FDR control

Pixel-wise analyses were performed over the recorded field of view. To focus inference on stimulus-responsive tissue and avoid diluting statistical power by including nonresponsive background pixels, hypothesis testing was restricted to an a priori response mask defined from control data for each condition. Specifically, for each stimulation condition (high or low stimulation, with or without gabazine), we computed the control mean map of the relevant response metric (PEAK for Fig. 2; SUM for Fig. 3) and defined the mask as pixels exceeding 50% of the maximum control value within the full field of view for that condition. Within this mask, group-level comparisons across four conditions (control, BPA, BB, and MB) were performed independently at each pixel using one value per slice (slice as the unit of replication; n equals the number of slices) with one-way ANOVA followed by Tukey's HSD. For each control-versus-compound comparison (control vs BPA, control vs BB, and control vs MB), the resulting *p* values were adjusted for multiple comparisons across pixels using the BH-FDR procedure applied across pixels within the mask to yield BH-FDR-adjusted *q* value maps (*q*-maps). Pixel-wise statistical maps are shown as *q*-maps thresholded at q<0.05. Corresponding uncorrected pixel-wise *p*-maps are provided in Extended Data Figure 2-1.

#### Slice-averaged maps and ROI averages

For visualization, PEAK and SUM metric maps were averaged across slices within each condition (n=4−6) to obtain slice-averaged maps. For ROI averages, PEAK and SUM values were averaged within anatomically defined ROIs (CA1, CA3, and DG) for each slice (one value per ROI per slice per condition). The primary dataset comprised 22 slices from six animal/days at each age (2 weeks, control/BPA/BB/MB = 6/5/5/6 slices; 8 weeks, 6/6/4/6 slices). For each prespecified age × ROI × stimulation intensity × inhibitory state × response metric stratum, ROI averages were analyzed with an animal/day-blocked linear model containing animal/day and treatment as categorical factors. The omnibus treatment effect was evaluated by type II ANOVA. Model-based pairwise contrasts among control, BPA, BB, and MB were adjusted by Tukey's method across all six treatment pairs within that stratum; only treatment-versus-control contrasts are marked in Figures 2–4. No additional correction was applied across the 48 prespecified strata. Residual normality was assessed with the Shapiro–Wilk test and homogeneity of variance across treatment groups with the median-centered Levene test. Complete omnibus statistics, degrees of freedom, effect estimates, simultaneous 95% confidence intervals, Tukey-adjusted *p* values, and assumption checks are provided in Extended Data Figure 2-2.

#### Handling of incomplete datasets

Not all slices yielded complete datasets for all conditions. For each analysis, slices were included if the required condition(s) for that specific comparison were available and met quality requirements. Consequently, sample size (*n*) may differ across conditions and comparisons; *n* is reported for each condition and comparison.

### Code accessibility

The custom Igor Pro procedures used for ROI extraction and pixel-wise statistics, together with the Python code and anonymized derived ROI data used for the animal/day-blocked revision analysis, are provided as Extended Data 1 under the MIT License (release version 0.1.0). The package also contains software requirements, expected numerical results, and verification tests. The original optical analysis was performed with Igor Pro 9 on macOS, and the revision analysis was validated with Python 3.11 on macOS.

10.1523/ENEURO.0165-26.2026.d1 Data 1 **Source code and derived ROI data used for the BPA VSD imaging analysis.** The ZIP archive contains the relevant Igor Pro functions extracted from the original analysis experiment, the Python implementation of the animal/day-blocked ROI analysis and BH–FDR adjustment, the Extended Data Figure 4-1 layout utility, anonymized slice-level ROI values, numerical reference results, and verification scripts. Download Data 1, ZIP file.

## Results

### Effect of BPA and its analogs (BB and MB) on hippocampal activity (PEAK)

We investigated the acute effects of BPA, BB, and MB across the three primary pathways of the hippocampal trisynaptic circuit: the Schaffer collateral projection to CA1, the mossy fiber projection to CA3, and the perforant pathway to DG. PEAK maps represent the spatial distribution of peak-response amplitude, defined as the maximum $\Delta F/F_{0}$ within 0–400 ms after stimulation. For visualization, PEAK maps were averaged across n=4−6 slices for each condition. Pixel-wise statistical results are shown as BH-FDR-adjusted *q*-maps thresholded at q<0.05 (Fig. 2*A*,*B*), whereas ROI-averaged PEAK values provide one average per ROI and slice (Fig. 2*C*,*D*). These two analyses were used as complementary readouts: *q*-maps localized FDR-controlled response differences within the evoked response field, whereas ROI averages quantified region-averaged response magnitude. After ROI-level comparisons were evaluated using the animal/day-blocked model and Tukey-adjusted contrasts, the significant ROI-level differences broadly paralleled the major patterns observed in the BH-FDR-corrected *q*-maps, while the *q*-maps further indicated where within the response fields these differences were spatially expressed. All preprocessing steps and analysis windows were identical across conditions.

**Figure 2.**
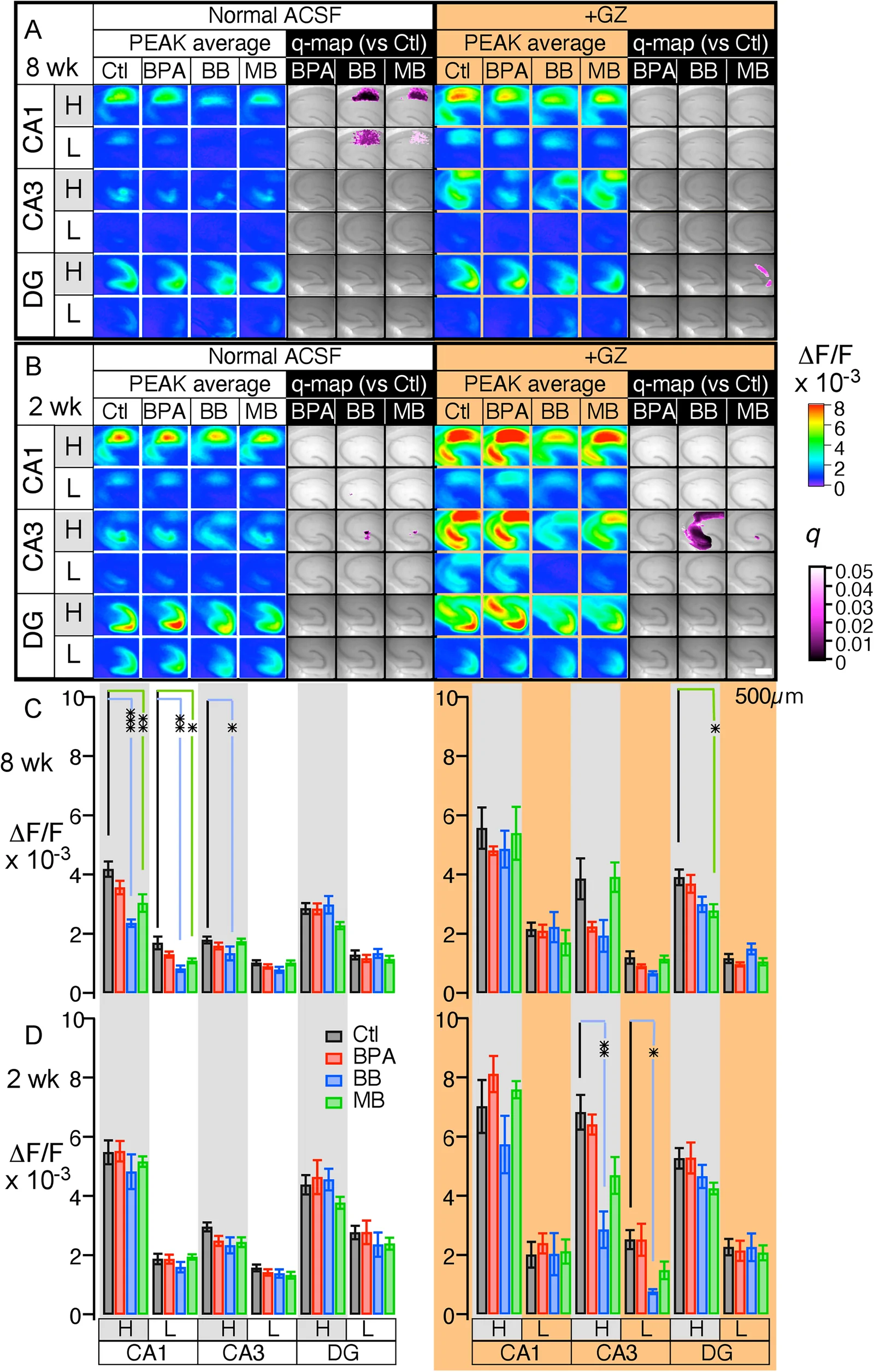
Pixel-wise statistical maps and ROI averages of stimulus-evoked VSD responses across hippocampal subregions. ***A, B***, Peak VSD response maps ( $\Delta F/F_{0}$, color; scale at right, $\times 10^{-3}$) for CA1, CA3, and the DG are shown for slices from 8 week (***A***) and 2 week (***B***) mice under high (H) and low (L) stimulation. Scale bar, 500 µm; the same spatial scale applies to all maps in ***A*** and ***B***. For each compound (BPA, BB, and MB), grayscale panels show pixel-wise *q*-maps for control-versus-compound comparisons, thresholded at q<0.05 after BH-FDR correction across pixels within the control-defined response mask. Normal ACSF (left) and gabazine (+GZ; right) conditions are shown. Pixel-wise statistics were computed using slice-level data at each pixel location, with BH-FDR correction applied within an a priori response mask defined from the control peak-response map for each condition (pixels exceeding 50% of the control maximum within the full field of view). ***C, D***, ROI-averaged PEAK values (mean maximum $\Delta F/F_{0}$ within anatomically defined CA1, CA3, and DG ROIs; one value per slice) for 8 week (***C***) and 2 week (***D***) groups under normal ACSF and +GZ. Bars show mean ± SEM ( n=4−6 slices per condition; six animal/days per age). ROI averages were analyzed separately for each age, ROI, stimulation intensity, inhibitory state, and metric using an animal/day-blocked model, followed by Tukey-adjusted pairwise contrasts across all six treatment pairs. Brackets show significant treatment-versus-control contrasts: ^*^p < 0.05, ^**^p < 0.01, and ^***^p < 0.001. Uncorrected pixel-wise *p*-maps are provided in Extended Data Figure 2-1, and complete ROI statistics are provided in Extended Data Figure 2-2.

10.1523/ENEURO.0165-26.2026.f2-1 Figure 2-1 ***Uncorrected pixel-wise*** p***-maps corresponding to the BH–FDR–adjusted*** q***-maps shown in Figures 2 and 3.*** *(A, B) PEAK and (C, D) SUM* p*-maps for 8-week (A, C) and 2-week (B, D) slices, shown for the same conditions as in Figures 2–3 (normal ACSF vs. +GZ; high and low stimulation; CA1/CA3/DG). Scale bar, 500* μ*m; the same spatial scale applies to all maps. For each compound (BPA, BB, and MB), uncorrected pixel-wise* p*-values are shown for control-versus-compound comparisons. Colored pixels indicate uncorrected* p<0.05*; the reversed viridis scale assigns brighter yellow to lower* p *values and darker purple to values approaching 0.05, while pixels with* p≥0.05 *are left uncolored over the grayscale anatomical reference. At each pixel, group-level comparisons among control, BPA, BB, and MB were performed using one-way ANOVA followed by Tukey’s HSD to obtain pixel-wise* p*-values for each control-versus-compound comparison. BH–FDR correction was applied across pixels within the control-defined response mask for each condition (Ctl response* >50% *of the control maximum in the full field of view; see Methods). The corresponding BH–FDR–adjusted* q*-maps are shown in Figures 2 and 3*. Download Figure 2-1, TIF file.

10.1523/ENEURO.0165-26.2026.f2-2 Figure 2-2 **Complete ROI statistics for the animal/day-blocked analysis.** The workbook contains group-level descriptive statistics, complete type-II ANOVA results for all 48 prespecified strata, residual degrees of freedom and effect sizes, Shapiro–Wilk and median-centered Levene assumption checks, and all six Tukey-adjusted pairwise contrasts with simultaneous 95% confidence intervals for each stratum. Download Figure2-2, XLS file.

### Pathway-specific reductions in young-adult mice (8 weeks)

In young-adult slices under normal ACSF, BB, and MB reduced PEAK responses most prominently in CA1. ROI-averaged PEAK values showed significant reductions in CA1 after BB and MB exposure under both high (H) and low (L) stimulation, together with an additional BB-associated reduction in CA3 under high stimulation (Fig. 2*C*). The corresponding BH-FDR-corrected *q*-maps showed FDR-controlled response differences within the CA1 response field for BB and MB (Fig. 2*A*). Thus, the ROI averages and *q*-maps converged on CA1 as the principal site of BB- and MB-associated PEAK reduction under normal ACSF, while the *q*-maps further showed that these differences were expressed within restricted portions of the CA1 response field rather than uniformly across the entire anatomical ROI. In contrast, BPA produced no comparable ROI-level or *q*-map-detectable PEAK reduction under normal ACSF.

Gabazine (10 µM) markedly changed the pattern of compound-associated PEAK reductions. The CA1 reductions observed under normal ACSF were no longer evident in the ROI averages after gabazine application (Fig. 2*C*). Instead, the statistically supported ROI-level effect was restricted mainly to the DG response under high stimulation, where MB reduced PEAK amplitude relative to control. Consistent with this ROI-level result, the corresponding *q*-map showed a spatially restricted FDR-controlled signal in the DG response field for MB under high stimulation (Fig. 2*A*). These results indicate that pharmacological blockade of GABA*_A_* receptor-mediated inhibition attenuated the CA1-dominant BB/MB effect observed under normal ACSF and shifted the detectable young-adult PEAK phenotype toward a more limited DG-associated effect. Overall, in young-adult slices, ROI averages and *q*-maps provided consistent support for the major PEAK effects, while *q*-maps added spatial information about where within the evoked response fields the statistically supported differences were expressed.

### Smaller and state-dependent effects in juvenile mice (2 weeks)

In juvenile slices under normal ACSF, compound-associated PEAK changes were limited. ROI-averaged PEAK values did not show clear reductions across CA1, CA3, or DG for any compound (Fig. 2*D*). The corresponding BH-FDR-adjusted *q*-maps showed only small, spatially restricted signals in the CA3 response field for BB and MB under high stimulation (Fig. 2*B*).

After gabazine application, compound-associated PEAK changes became more evident in CA3. ROI averages showed a CA3-centered PEAK reduction for BB, whereas the *q*-maps revealed broader spatial involvement: the BB-associated signal under high stimulation extended across the CA3 response field and into adjacent CA1, while the MB-associated signal remained restricted to a small stimulation-proximal region within CA3 (Fig. 2*B*,*D*). Overall, juvenile PEAK effects were modest under normal ACSF but became more detectable under gabazine, particularly for BB in the CA3-centered response field.

### Impact on response duration and integrated activity (SUM)

To assess compound effects on integrated circuit responses, we analyzed the response integral (SUM), defined as the time integral of the $\Delta F/F_{0}$ signal over the fixed poststimulus window (0–400 ms). SUM captures both response amplitude and duration within this window. Pixel-wise statistical results are shown as BH-FDR-adjusted *q*-maps thresholded at q<0.05 (Fig. 3*A*,*B*), and ROI-averaged SUM values provide one average per ROI and slice (Fig. 3*C*,*D*). As for PEAK, ROI averages quantify region-averaged activity, whereas *q*-maps localize SUM differences that survived pixel-wise FDR correction.

#### Young-adult mice (8 weeks)

In young-adult slices under normal ACSF, ROI-averaged SUM values indicated reduced integrated activity in CA1 after BB and MB exposure, together with a BPA-associated reduction under high stimulation (Fig. 3*C*). The corresponding BH-FDR-adjusted *q*-maps showed FDR-controlled SUM differences mainly within the CA1 response field, with signals detected for BB under both stimulation levels but little or no detectable signal for BPA or MB (Fig. 3*A*). This dissociation indicates that ROI-level SUM reductions were not always accompanied by spatially localized FDR-controlled effects.

**Figure 3.**
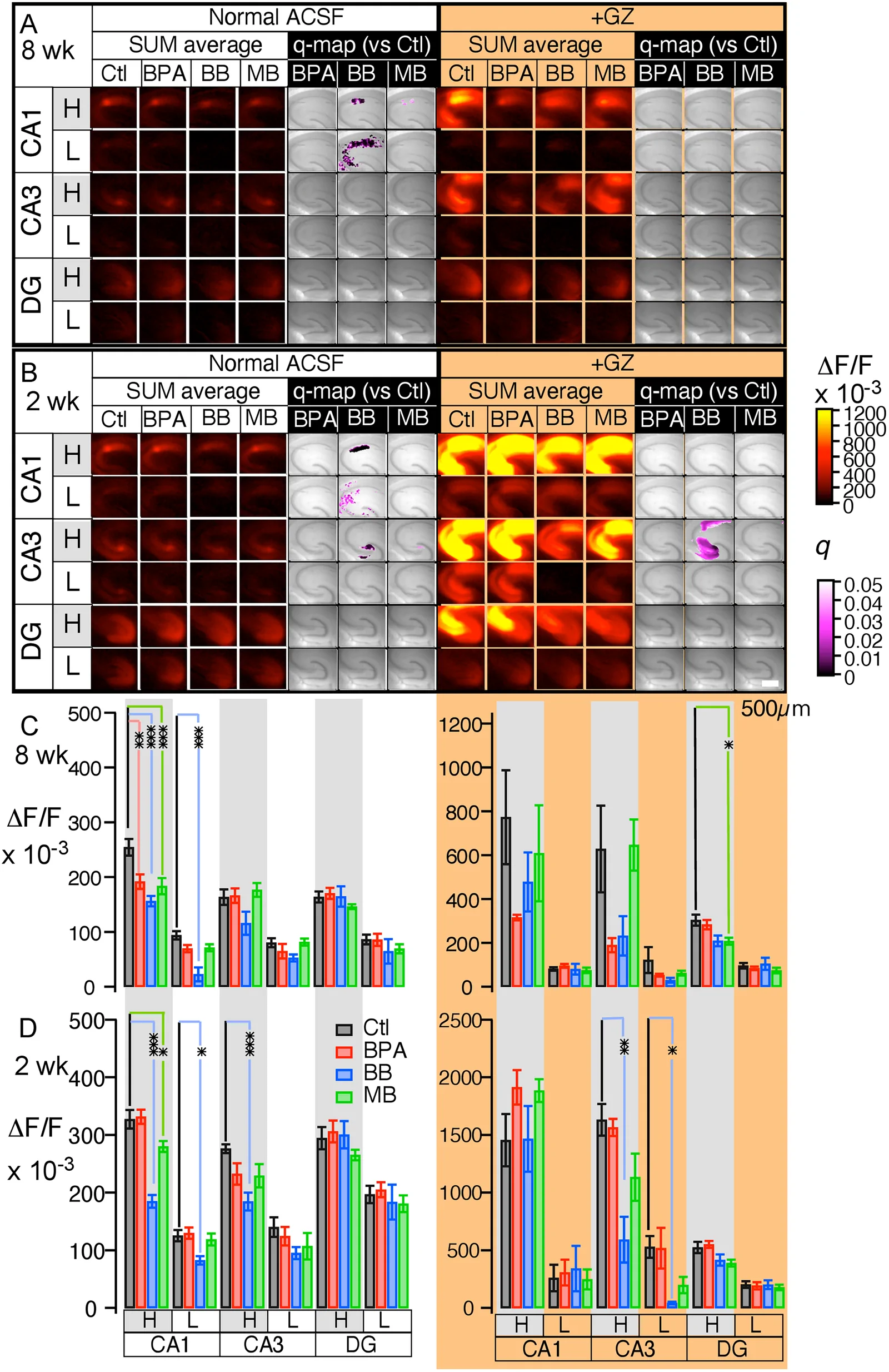
Pixel-wise statistical maps and ROI averages of stimulus-evoked VSD responses across hippocampal subregions based on the SUM metric. ***A, B***, SUM maps (time integral of $\Delta F/F_{0}$ over 0–400 ms, color; scale at right) for CA1, CA3, and the DG are shown for slices from 8 week (***A***) and 2 week (***B***) mice under high (H) and low (L) stimulation. Scale bar, 500 µm; the same spatial scale applies to all maps in ***A*** and ***B***. For each compound (BPA, BB, and MB), grayscale panels show pixel-wise *q*-maps for control-versus-compound comparisons, thresholded at q<0.05 after BH-FDR correction across pixels within the control-defined response mask. Normal ACSF (left) and gabazine (+GZ; right) conditions are shown. Pixel-wise statistics were computed using slice-level data at each pixel location, with BH-FDR correction applied within an a priori response mask defined from the control SUM map for each condition (pixels exceeding 50% of the control maximum within the full field of view). ***C, D***, ROI-averaged SUM values (mean within anatomically defined CA1, CA3, and DG ROIs; one value per slice) for 8 week (***C***) and 2 week (***D***) groups under normal ACSF and +GZ. Bars show mean ± SEM ( n=4−6 slices per condition; six animal/days per age). ROI averages were analyzed separately for each age, ROI, stimulation intensity, inhibitory state, and metric using an animal/day-blocked model, followed by Tukey-adjusted pairwise contrasts across all six treatment pairs. Brackets show significant treatment-versus-control contrasts: ^*^p < 0.05, ^**^p < 0.01, and ^***^p < 0.001. Uncorrected pixel-wise *p*-maps are provided in Extended Data Figure 2-1, and complete ROI statistics are provided in Extended Data Figure 2-2.

After gabazine application, the CA1-centered SUM reductions observed under normal ACSF were no longer detected in the ROI averages. Instead, ROI-averaged SUM was reduced in DG under high stimulation after MB exposure (Fig. 3*C*). The corresponding *q*-maps showed little or no detectable FDR-controlled signal under gabazine (Fig. 3*A*), indicating that this DG-associated ROI-level reduction was not accompanied by a clearly localized pixel-wise effect.

#### Juvenile mice (2 weeks)

In juvenile slices under normal ACSF, ROI-averaged SUM values were reduced predominantly after BB exposure, with effects in CA1 under both stimulation levels and in CA3 under high stimulation (Fig. 3*D*). An additional MB-associated reduction was detected in CA1 under high stimulation, whereas no ROI-level SUM reduction was detected for BPA. The corresponding BH-FDR-adjusted *q*-maps showed spatially restricted BB-associated signals: under high stimulation, the signal was limited to part of the CA1 response field, whereas under low stimulation, it extended across CA1 and into CA3 (Fig. 3*B*).

After gabazine application, ROI-level SUM reductions remained BB-selective but were concentrated in CA3 under both stimulation levels (Fig. 3*D*). The corresponding *q*-maps showed a broad BB-associated FDR-controlled signal under high stimulation, extending across the CA3 response field and into adjacent CA1 (Fig. 3*B*). No clear ROI-level or *q*-map signal was detected for BPA or MB under gabazine.

Overall, juvenile SUM effects were predominantly BB-associated, with an isolated MB effect in CA1 under normal ACSF, and shifted toward a more CA3-centered BB pattern after gabazine application. ROI averages identified the affected subfields, whereas *q*-maps showed the spatial extent of BB-associated FDR-controlled effects when detectable.

### Overall assessment of circuit sensitivity to bisphenol analogs

To summarize ROI-based response patterns across subregions, stimulation levels, inhibitory conditions, and response metrics, we generated heatmaps of normalized PEAK and SUM values (Fig. 4). For each compound condition, ROI-averaged values were normalized to the corresponding control condition (control, 1.0). These heatmaps provide a compact overview of region-averaged response magnitude and should be interpreted together with the *q*-maps in Figures 2 and 3, which localize FDR-controlled pixel-wise effects.

**Figure 4.**
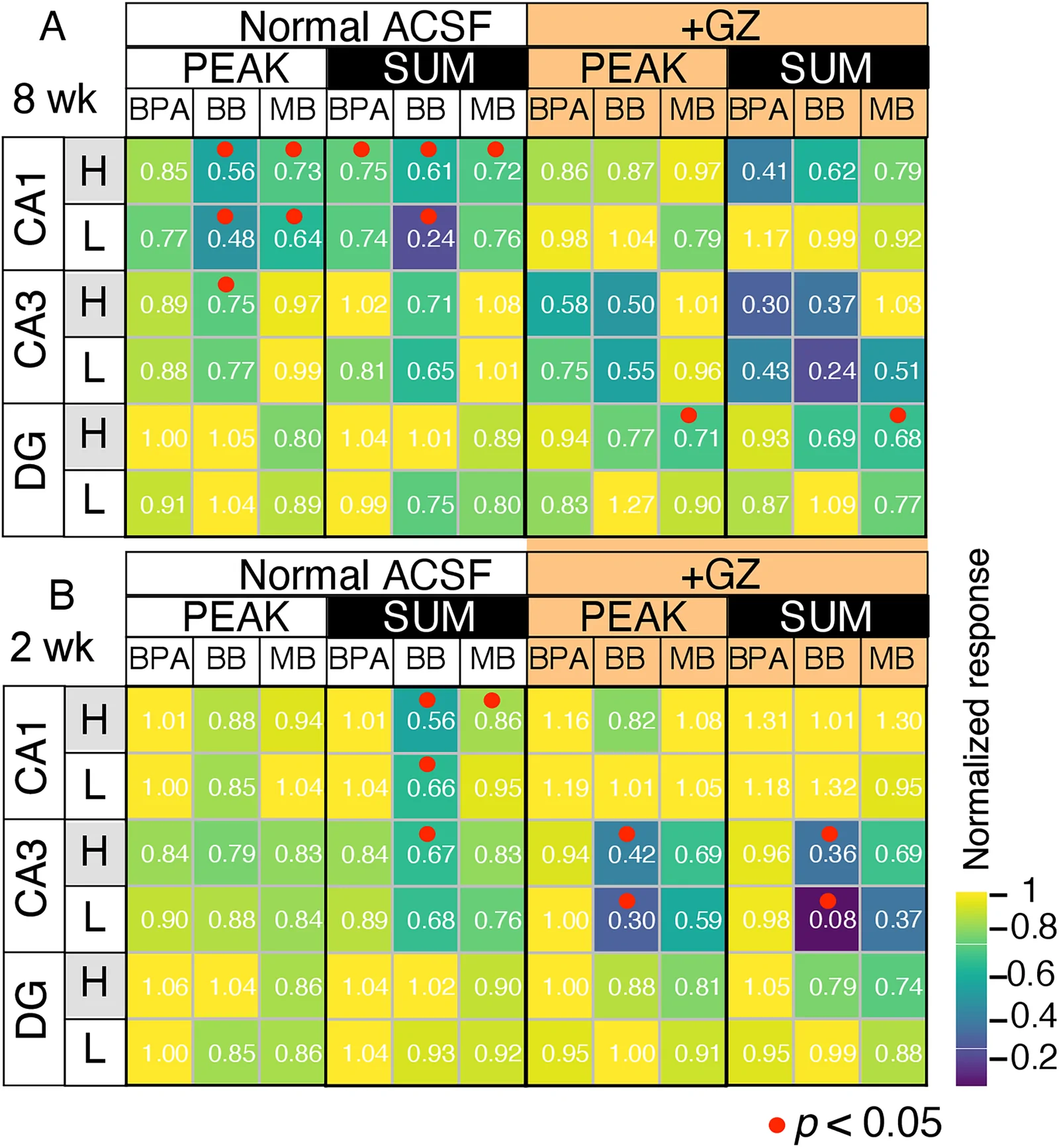
Heatmap summary of ROI-based hippocampal circuit responses across conditions. Heatmaps summarize normalized ROI-averaged PEAK and SUM responses relative to the corresponding control condition (control, 1.0) in 8 week (***A***) and 2 week (***B***) mice under normal ACSF and gabazine (+GZ). Rows indicate hippocampal subregions and stimulation intensities (CA1-H, CA1-L, CA3-H, CA3-L, DG-H, and DG-L), and columns indicate compounds (BPA, BB, and MB). Cell color and overlaid values represent normalized response magnitude. Red dots indicate significant treatment-versus-control contrasts from the animal/day-blocked model after Tukey adjustment across all six treatment pairs within each stratum (p<0.05). This figure summarizes the ROI-based results shown in Figures 2 and 3. The corresponding group-average temporal traces are provided in Extended Data Figure 4-1, and complete ROI statistics are provided in Extended Data Figure 2-2.

10.1523/ENEURO.0165-26.2026.f4-1 Figure 4-1 ***Group-average VSD response traces across all experimental conditions.*** *Traces show the group mean* $\Delta F/F_{0}$ *response for control (black), BPA (red), MB (green), and BB (blue), with shaded bands indicating SEM. Rows show CA1, CA3, and DG, and columns show low (L) and high (H) stimulation under normal ACSF and gabazine (+GZ). The dotted horizontal line in each panel indicates zero. The upper and lower blocks show 8-week and 2-week groups, respectively. For this descriptive trace display,* $F_{0}$ *was calculated from frames 30–58 before the stimulus (stimulus frame 62 at 1 ms/frame); group-average image sequences were processed with one pass of the Igor Pro* *ImageFilter gauss3d* *filter using a* 7×7 *kernel, without drift removal. These display-oriented settings did not replace the primary PEAK/SUM preprocessing (Methods), and comparison with the primary preprocessing showed no material change in the treatment-associated patterns. All panels use a shared display scale to facilitate comparison of response magnitude and time course across conditions. The traces are presented as a descriptive temporal comparison; although condition-dependent differences in response magnitude and decay are apparent, a large, consistent treatment-associated displacement of peak time is not evident across the complete dataset*. Download Figure 4-1, TIF file.

#### Overall patterns in young-adult mice (8 weeks)

In young-adult slices, the heatmaps showed more consistent ROI-level reductions after BB and MB exposure than after BPA exposure (Fig. 4*A*). Under normal ACSF, statistically supported reductions were concentrated mainly in CA1 for BB and MB and were observed in both PEAK and SUM; BB also reduced CA3 PEAK under high stimulation, and an isolated BPA-associated CA1 SUM reduction was detected under high stimulation. After gabazine application, the CA1-centered pattern was no longer dominant; statistically supported reductions were restricted to DG under high stimulation after MB exposure for both PEAK and SUM. Overall, the 8 week heatmaps indicate that BB and MB produced broader ROI-level effects than BPA and that inhibitory state shifted the dominant affected region from CA1 under normal ACSF toward DG under gabazine.

#### Overall patterns in juvenile mice (2 weeks)

In juvenile slices, statistically supported ROI-level reductions were more selective than in young adults (Fig. 4*B*). Under normal ACSF, reductions were detected mainly after BB exposure, particularly in SUM, with one additional MB-associated CA1 SUM reduction under high stimulation; BPA showed no statistically supported reduction. After gabazine application, the clearest ROI-level pattern remained BB-associated and was centered mainly on CA3, again most evident in SUM. Thus, unlike the broader BB/MB-associated pattern observed in young-adult slices, juvenile effects were more restricted to specific compound, metric, and inhibitory state combinations.

Overall, the heatmaps emphasize that phenolic analog effects were broader than BPA-associated effects in young-adult slices but more selective and mainly BB-associated in juvenile slices.

Group-mean traces showed no large, consistent treatment-associated difference in peak timing across the examined conditions (Extended Data Fig. 4-1).

## Discussion

The primary goal of this study was to develop and validate an integrated VSD imaging analysis pipeline for the spatially resolved comparison of acute circuit responses. The hippocampal trisynaptic circuit provides a well-characterized, tractable preparation for this purpose, as VSD imaging effectively captures fast, spatially extended population membrane potential dynamics across the CA1, CA3, and DG subfields (Cohen et al., 1978; Grinvald and Hildesheim, 2004; Knöpfel and Song, 2019). While previous studies have demonstrated the utility of VSD imaging for assessing hippocampal and cortical dynamics (Tominaga et al., 2018; Utsumi et al., 2023), robust quantitative comparison across experimental conditions remains a challenge due to the high spatiotemporal complexity and interslice variability of VSD signals. Addressing this limitation requires analytical procedures that can simultaneously summarize regional response magnitudes, visualize global spatial organization, and appropriately treat the biological receptacle (the brain slice)—rather than individual pixels—as the independent unit of replication, thereby avoiding pseudoreplication and inflated statistical support.

We applied this pipeline to a demonstration dataset evaluating the acute effects of BPA and two structurally related phenolic analogs, BB and MB. These alternatives were selected due to rising concerns over the “regrettable substitution” of BPA (Trasande, 2017; Pelch et al., 2019) and growing evidence that such analogs alter neural or behavioral endpoints (Palanza et al., 2008; Sasaki et al., 2023). The pipeline successfully decoupled regional response magnitude from spatial expression, revealing subtle, spatially nonuniform, and condition-dependent circuit effects. Within this dataset, the phenolic analogs induced more detectable suppressions of circuit responses than BPA, with these outcomes highly modulated by pathway, age, response metric, and baseline inhibitory state. These findings illustrate the central utility of our framework: it uncovers complex circuit phenotypes that would remain obscured by single-site electrophysiology or conventional ROI averaging alone.

### Multiscale spatial and statistical interpretation of VSD signals

A core methodological contribution of this framework is the parallel integration of ROI averages, uncorrected pixel-wise *p*-maps, and BH-FDR-controlled *q*-maps. Rather than acting as competing statistical tests, these readouts operate across complementary spatial and statistical scales. ROI averages quantify the mean response magnitude within anatomically defined subfields, maximizing statistical power for regional comparisons. Uncorrected *p*-maps preserve and visualize the nominal spatial topology of the evoked activity, acting as a technically informative spatial summary. Finally, BH-FDR-controlled *q*-maps rigorously isolate the specific pixel clusters where compound-associated effects survive correction for multiple comparisons.

This multilevel approach is vital for functional optical imaging, where neural population activity propagates across broad fields of view. Compound-associated perturbations may be highly localized or, conversely, spatially coherent but too weak to survive conservative pixel-wise FDR thresholds. For example, a spatially restricted effect may emerge clearly on a *q*-map yet contribute minimally to an ROI average if it spans only a small fraction of that subfield. Conversely, widespread subtle shifts may register in ROI averages but fail to pass pixel-wise thresholding. Furthermore, because imaging data exhibit high spatial autocorrelation and are sensitive to preprocessing steps such as spatial smoothing, *q*-maps are optimally interpreted as spatial localization summaries rather than rigid anatomical boundaries of affected microdomains.

### Distinct acute circuit phenotypes of BPA-related phenolic analogs

The demonstration dataset establishes that BPA and its structurally related phenolic analogs, BB and MB, produce distinct acute hippocampal circuit phenotypes. Under the tested ex vivo conditions, BB and MB drove more robust, detectable reductions in evoked population responses than the parent compound, BPA. This divergence does not imply that BPA is biologically inert in this system; rather, it demonstrates that within the parameters of this acute assay, BPA-associated suppressions were limited, whereas its structural analogs exerted clear circuit-level target engagement. These results underscore the necessity of empirically profiling replacement compounds rather than assuming toxicological equivalence based on structural similarity (Sasaki et al., 2023).

The observed response reductions match reports that specific bisphenol alternatives attenuate excitatory synaptic transmission (Bi et al., 2021, 2025). However, population-level VSD imaging evaluates integrated membrane potential dynamics and cannot inherently resolve the precise cellular or subcellular locus of action (Knöpfel and Song, 2019). The observed circuit-level suppression could stem from diminished presynaptic glutamate release, altered postsynaptic receptor currents, enhanced local interneuron recruitment, or depressed intrinsic membrane excitability. The utility of the present pipeline is therefore not to isolate a single molecular mechanism but to successfully differentiate compound-specific circuit phenotypes as a function of experimental condition.

### Age-dependent sensitivity and network-state constraints

The juvenile dataset highlights a critical distinction between immediate circuit sensitivity to acute chemical exposure and cumulative developmental vulnerability. Although developing neural networks are highly sensitive to environmental insults, juvenile slices in this acute assay did not exhibit uniform hypersensitivity. Instead, xenobiotic effects in the juvenile hippocampus were highly selective, predominantly associated with BB with an isolated MB-associated CA1 SUM effect, and strongly dependent on the chosen response metric and baseline inhibitory state.

This age-specific selectivity does not indicate developmental resistance to phenolic analogs. Early-life exposure designs typically evaluate cumulative toxicity across critical windows of differentiation, synaptogenesis, and network maturation, which manifest as delayed behavioral deficits in adulthood (Sasaki et al., 2023). In contrast, our assay captures immediate population responses to acute exposure in isolated ex vivo networks. Evoked responses in the juvenile network may be buffered or masked by the unique biophysical properties of the immature circuit. During the second and third postnatal weeks, the rodent hippocampus undergoes the final phases of the GABAergic switch—where GABAergic signaling transitions from depolarizing to hyperpolarizing—alongside the continuous maturation of recurrent inhibition and network gating (Garaschuk et al., 1998; Ganguly et al., 2001; Ben-Ari, 2002; Ben-Ari et al., 2007). This highly dynamic network state provides a plausible neurodevelopmental context for the distinct acute phenotypes observed between 2-week-old and 8-week-old preparations, confirming that acute pharmacological sensitivity and cumulative developmental vulnerability represent distinct experimental endpoints.

The network-state dependence observed during $\mathrm{GAB}A_{A}$ receptor blockade provides further mechanistic constraints. The circuit profiles of BB and MB were not fixed, intrinsic properties of the chemicals alone but were fundamentally shaped by baseline inhibitory tone. In young-adult slices, the CA1-centered suppression observed under normal ACSF was attenuated by gabazine treatment, whereas juvenile CA3-centered effects became unmasked and more pronounced under gabazine, particularly for BB. This pattern suggests that the spatial manifestation of compound toxicity is tightly coupled to the local excitation–inhibition (E/I) balance, likely involving differential actions on recurrent collaterals or cell-type-specific interneuron networks (Menzikov et al., 2024).

This multicomponent action is supported by the divergent patterns observed between the PEAK and SUM metrics. While PEAK captures maximal population response amplitude, SUM integrates activity over a wide temporal window (0–400 ms), reflecting both initial amplitude and response duration. The finding that juvenile perturbations were more evident in the SUM metric suggests that acute analog exposure may preferentially alter the persistence or integrated output of population activity rather than simply dampening initial peak amplitudes.

### Methodological limitations and future directions

Several scope conditions apply to the interpretation of these findings. First, the lower number of statistically supported effects for BPA must not be construed as evidence of safety; this outcome is strictly bounded by the acute timing, specific concentrations, and statistical thresholds of the current protocol. Second, while ex vivo slices are ideal for high-throughput, highly controlled circuit perturbations, they do not recapitulate in vivo pharmacokinetics, blood–brain barrier penetration, hepatic metabolism, or chronic, low-dose cumulative exposure.

Third, because the axial sensitivity of the wide-field epifluorescence measurement was not calibrated, the recorded population responses cannot be assigned to a specific axial depth or cell layer. Fourth, resolving the precise synaptic and cellular mechanisms underlying these population-level shifts will require complementary single-cell or pathway-specific approaches, such as patch-clamp recordings, targeted field potentials, or high-speed, high-resolution optical quantifications of single cells. Fifth, pixel-wise inference remains sensitive to spatial autocorrelation and preprocessing configurations. Although the analysis window and smoothing parameters were standardized here to eliminate bias, future iterations could incorporate spatially informed cluster-level statistics, effect-size mapping, or spatial hierarchical models to enhance the detection of weak, spatially coherent signals.

Sixth, the slice was retained as the treatment-level unit of observation and animal/day was included as a blocking factor to account for the shared animal origin of slices. Scaling the design to larger animal cohorts would nevertheless permit more flexible mixed-effects modeling and more precise partitioning of animal-level and slice-level variance. Finally, this validation was conducted exclusively in male mice. Given that bisphenol-related compounds are established endocrine disruptors that interact with estrogen receptor signaling pathways, these acute circuit phenotypes cannot be assumed to generalize across sexes. Future studies must incorporate female cohorts and be powered to evaluate explicit sex-by-compound, sex-by-age, and sex-by-inhibitory state interactions.

## Conclusion

This study developed and validated an integrated VSD imaging analysis framework for quantitative, spatially resolved comparisons of acute hippocampal circuit responses while preserving the brain slice as the independent unit of replication. By combining ROI averages, uncorrected pixel-wise *p*-maps, and BH-FDR-controlled *q*-maps, this multiscale approach distinguished region-averaged response magnitudes from the nominal and FDR-controlled spatial patterns of compound-associated effects. In an acute slice assay using BPA and its phenolic analogs, BB and MB, analog-associated reductions in response magnitude were strongly modulated by compound identity, developmental age, inhibitory network state, response metric, and hippocampal subfield. Specifically, BB and MB produced more consistent, statistically supported suppression than the parent compound BPA in young-adult slices, whereas juvenile responses were highly selective and showed prominent BB-associated alterations only under specific PEAK and SUM conditions. ROI averages and *q*-maps provided complementary insights: ROI analysis quantified global, region-averaged response reductions, whereas *q*-maps localized the spatial expression of FDR-controlled pixel-wise differences when such effects were detectable. Taken together, these results establish a practical and reproducible analytical pipeline for comparative circuit-level phenotyping of chemical or environmental perturbations under standardized ex vivo conditions.

## Synthesis

Reviewing Editor: Matthew Grubb, King's College London Decisions are customarily a result of the Reviewing Editor and the peer reviewers coming together and discussing their recommendations until a consensus is reached. When revisions are invited, a fact-based synthesis statement explaining their decision and outlining what is needed to prepare a revision will be listed below. The following reviewer(s) agreed to reveal their identity: Thomas Knopfel.

Both Reviewers have generally positive views on the manuscript, but also request revisions to better support the paper's conclusions. Please directly address all of the below comments in any resubmission.

Reviewer 1:

Stimulation-induced neuronal responses imaged with voltage-sensitive dyes (VSD) have traditionally been analysed by comparing spatial averages across regions of interest (ROs). A caveat of this approach is the often somewhat arbitrarily defined ROI outlines. This issue is addressed by the approach presented in present report where statistical analysis is performed on pixel-by-pixel bases, revealing regions of significant difference which then can be associated with anatomical landmarks. The authors applied this approach (analysis pipeline) to compare the acute effects of bisphenol A (BPA) BPA with those of two structurally related phenolic analogues.

The paper is well written and figures are well prepared.

Conclusions are justified by the data and limitations are well considered.

Minor:

While the term "ROI summaries" is well understandable from the context, I suggest using the better comprehendible term ROI averages (introduced as averages across ROIs). Similarly, terms like Response Amplitudes and Response Integrals may be easier to catch than PEAK and SUM (which sound a bit like lab slang). However, this is a matter of taste and my opinion on this is not strong.

Reviewer 2:

This study proposed an analytical pipeline for voltage-sensitive dye imaging in brain slice. Using the imaging and analytical method, the authors found that BPA and its two phenolic analogs altered acute evoked responses in the hippocampal circuits in young adult and juvenile mice. Overall, the experiments are well designed, the statistics are generally appropriate, the conclusions are supported by the results. However, the manuscript could be improved by adding more complete statistical results. I have following questions and suggestions:

1. What is the depth range of activities that can be detected by voltage-sensitive dye imaging in slice?

2. What is the imaging resolution. What is the actual dimension of one pixel?

3. How did the authors determine the sample size in this recording and analytical method? In adult gabazine group, CA3 response with BPA and BB treatment reduced but has no significant differences. Could this be due to insufficient statistical power?

4. Among the regions and treatments, is there any difference observed in peak response latency or response slope?

5. In the ROI statistics, please include more details of ANOVA tests. Did the data pass normality and equal variance test before proceeding to ANOVA? And please report the full results of ANOVA tests, including the p value for main effect, interaction effect, and degrees of freedom.

6. Since this study proposed an analytical pipeline for VSD imaging, sharing the analysis code would greatly enhance the reproducibility and accessibility of the method. I encourage the authors to make the code publicly available, for example through a repository or as supplementary material.

7. In figure 1D, what does the blue and black trace mean respectively?

8. In figure 1F, the figure legend describes how the SUM maps were computed. However, the figure itself shows how the average maps were obtained. Please correct the discrepancy.

9. In supplementary figure 1C, the color scale of the p value is confusing. Please revise the color scheme to improve clarity.

## Author Response

Response to the Editor and Reviewers Manuscript eN-MNT-0165-26 Pixel-wise statistical mapping with voltage-sensitive dye imaging reveals differential hippocampal circuit effects of bisphenol A and related phenolic analogs Dear Dr. Grubb and Reviewers, We thank the Reviewing Editor and both Reviewers for their constructive and careful evaluation of our manuscript. We have organized the response below on a point-by-point basis. Newly added text is shown in bold type in the marked copy, and a separate clean copy is also provided. Page and line numbers below refer to the marked anonymous revised manuscript used for peer review.

Synthesis statement and editorial requirement Code Accessibility Comment: Detailed analysis of data is presented, so the authors need to provide infor mation on the code and software used in a specific "Code Accessibility" section of the Methods.

Response: We agree that explicit access to the analysis code and software is important for reproducibility. We added a dedicated Code Accessibility subsection to the Materials and Meth ods. The reproducibility package is provided as Extended Data 1 and identifies the relevant Igor Pro procedures, the revision-analysis scripts, software requirements, version information, and the MIT license.

Changes in the manuscript: A new "Code Accessibility" subsection was added to Materials and Methods (p. 9, lines 223-227).

Reviewer 1 Comment 1: Terminology Comment: While the term "ROI summaries" is well understandable from the context, I suggest using the better comprehensible term ROI averages (introduced as averages across ROIs). Similarly, terms like Response Amplitudes and Response Integrals may be easier to catch than PEAK and SUM.

Response: We thank the Reviewer for this helpful suggestion. We replaced "ROI summaries" with "ROI averages," introduced as values averaged within each anatomical ROI. In the Abstract, we now use the plain-language terms "peak amplitude" and "time integral." In Materials and Methods, we define the formal measures as "peak response amplitude (PEAK)" and "response 1 integral (SUM)." We retain PEAK and SUM thereafter, particularly in figure and table headings, to keep the labels compact and consistent across experimental conditions.

Changes in the manuscript: Terminology was revised consistently in the Abstract, Materials and Methods, Results, Discussion, Conclusion, and figure legends.

Reviewer 2 Comment 1: Detectable depth Comment: What is the depth range of activities that can be detected by voltage-sensitive dye imaging in slice? Response: We agree that the depth sensitivity should be stated more clearly. The 350 µm slice thickness should not be interpreted as the depth sampled uniformly by the epifluorescence measurement. The system does not provide optical sectioning, and dye staining was visibly weaker at the surface opposite the imaged surface. Moreover, the reported scattering mean free path for visible light in extracted brain gray matter is approximately 50-100 µm (Helmchen and Denk, 2005), while surface VSD measurements are generally weighted toward superficial tissue (Grinvald and Hildesheim, 2004). We therefore estimate that labeled membranes within approximately the superficial 50-100 µm made the dominant contribution to our signal. This is an order-of-magnitude estimate, not a directly calibrated cutoff; the precise axial weighting also depends on dye penetration, wavelength, tissue scattering, and collection geometry.

Changes in the manuscript: We added a brief statement to the VSD Imaging and Optical Recording subsection explaining that wide-field epifluorescence produces a depth-weighted pop ulation signal rather than a defined optical section. We also added a concise limitation stating that the present responses cannot be assigned to a specific axial depth or cell layer; the optical basis for the approximate depth range is provided in this response (p. 5, lines 127-128; p. 18, lines 449-451).

Comment 2: Imaging resolution and pixel dimension Comment: What is the imaging resolution? What is the actual dimension of one pixel? Response: We agree and now report the calibrated spatial sampling at the specimen plane.

Each image comprised 92 × 80 pixels. The physical sampling dimensions were 15.625 µm/pixel in the x direction and 16.13 µm/pixel in the y direction, giving a field of view of approximately 1.44 mm × 1.29 mm. These are the calibrated image-sampling dimensions. Because the optical point-spread function was not measured independently, we do not equate the pixel dimensions with a separately measured optical resolution. We also specified the manufacturer and model number of the lens used as the projection lens in the tandem-lens system.

2 Changes in the manuscript: We specified the projection lens and added the acquired im age dimensions, calibrated specimen-plane pixel dimensions, calculated field of view, and the distinction between image sampling and independently measured optical resolution to the VSD Imaging and Optical Recording subsection of Materials and Methods (p. 5, lines 121-126; p. 6, lines 129-135).

Comment 3: Sample size and statistical power Comment: Howdid the authors determine the sample size in this recording and analytical method? In the adult gabazine group, CA3 response with BPA and BB treatment reduced but has no significant differences. Could this be due to insufficient statistical power? Response: We agree that the experimental hierarchy, sample size, and uncertainty of the non significant comparisons should be described explicitly. No a priori power calculation was per formed; sample size was determined by the complete set of available experiments that met the prespecified quality requirements and by the practical feasibility of the slice-recording design.

One animal was used per experimental day, and treatment conditions were distributed across slices from that animal when feasible. The primary ROI dataset contained 22 slices from six animal/days at each age (2 weeks: control/BPA/BB/MB = 6/5/5/6 slices; 8 weeks: 6/6/4/6 slices). We therefore reanalyzed the ROI data with animal/day as a blocking factor and re tained Tukey adjustment across all six treatment pairs. The adult +GZ CA3 BPA and BB con trasts remained non-significant across high/low stimulation and PEAK/SUM (Tukey-adjusted p = 0.0901-0.4941); their simultaneous 95% confidence intervals included zero. We now avoid treating these results as evidence of absence and state that the available sample size does not exclude effects smaller than those detectable with the present design.

Changes in the manuscript: We added the sample-size rationale, animal/day and slice hi erarchy, group-specific sample counts, blocked-model specification, and uncertainty reporting to Materials and Methods and Extended Data Table 2-1 (p. 7, lines 165-173; p. 9, lines 202-222).

Comment 4: Peak latency and response slope Comment: Among the regions and treatments, is there any difference observed in peak response latency or response slope? Response: We thank the Reviewer for raising this temporal aspect of the responses. We gen erated group-average traces with SEM for CA1, CA3, and DG at both stimulation levels, ages, and bath conditions. The traces show condition-dependent differences in response magnitude and time course, but no large, consistent shift in peak time is evident across the full dataset. The fast stimulus-associated component is not sufficiently resolved to support formal latency or slope comparisons or to separate presynaptic and postsynaptic contributions. We therefore added the complete trace matrix as Extended Data Figure 4-1 as a descriptive temporal comparison without assigning a mechanistic interpretation to the earliest component.

3 A NormalACSF +GZ B NormalACSF +GZ 0 100 Time (ms) 0 10 0 100 Time (ms) 0 10 0 100 Time (ms) 0 10 0 100 Time (ms) 0 5 10 0 100 Time (ms) 0 5 10 0 100 Time (ms) 0 5 10 0 100 Time (ms) 0 5 10 0 100 Time (ms) 0 5 10 0 100 Time (ms) 0 5 10 0 100 Time (ms) 0 5 10 0 100 Time (ms) 0 5 10 0 100 Time (ms) 0 5 10 0 100 Time (ms) 0 0 100 Time (ms) 0 0 100 Time (ms) 0 0 100 0 5 10 0 100 Time (ms) 0 5 10 0 100 Time (ms) 0 5 10 0 100 Time (ms) 0 5 10 0 100 Time (ms) 0 5 10 0 100 Time (ms) 0 10 0 100 Time (ms) 0 10 0 100 Time (ms) 0 5 10 8wk NormalACSF +GZ NormalACSF +GZ Shadedband:SEM Ctl BPA MB BB L H L H L H L H CA1 CA3 DG CA1 CA3 DG 2wk 50ms 5x10-3 ∆F/F0 ExtendedDataFigure4-1. Group-averageVSDresponsetracesacrossall experimental condi tions.LinesshowgroupmeansandshadedbandsshowSEM.

4 Changes in the manuscript: We added the complete all-condition trace matrix as Extended Data Figure 4-1 and a concise statement in Results that no large, consistent treatment-associated difference in peak timing was evident across the examined conditions (p. 14, lines 346-347; Ex tended Data Figure 4-1, pp. 31-32).

Comment 5: Complete ANOVA results and assumptions Comment: In the ROI statistics, please include more details of ANOVA tests. Did the data pass normality and equal variance tests before proceeding to ANOVA? Please report the full results of ANOVA tests, including the p value for main effect, interaction effect, and degrees of freedom.

Response: We agree and have expanded the statistical reporting. For each prespecified age × ROI × stimulation-intensity × inhibitory-state × response-metric stratum, we fitted ROI mean ∼ animal/day + treatment. The treatment effect was evaluated by type-II ANOVA, followed by model-based pairwise contrasts with Tukey adjustment across all six treatment pairs. Because these were prespecified stratified models rather than a single factorial model, interaction terms do not arise within an individual model. We assessed residual normality with Shapiro-Wilk tests and homogeneity of variance with median-centered Levene tests. Three of 48 strata were flagged at p < 0.05 by each check, and these results are reported transparently rather than described as universally satisfying the assumptions. Extended Data Table 2-1 now provides the treatment and animal/day F statistics, numerator and residual degrees of freedom, exact p values, partial η2, assumption checks, all six Tukey-adjusted contrasts, and simultaneous 95% confidence intervals.

Changes in the manuscript: Materials and Methods, Results, the Figure 2-4 ROI panels and legends, and Extended Data Table 2-1 were updated to use the same animal/day-blocked Tukey analysis (p. 9, lines 202-217; Results, pp. 10-14; Figures 2-4, pp. 26-28).

Comment 6: Code sharing Comment: Since this study proposed an analytical pipeline for VSD imaging, sharing the analysis code would greatly enhance the reproducibility and accessibility of the method.

Response: We agree and have prepared a minimal reproducibility package as Extended Data 1.

It contains the relevant Igor Pro procedures, scripts used to produce the revision analyses, an anonymized derived ROI dataset, expected results, verification tests, software requirements, and the MIT license. This package contains the code and derived data required to reproduce the primary ROI and pixel-wise analyses.

Changes in the manuscript: A Code Accessibility subsection was added to Materials and Methods, and the reproducibility package is supplied as Extended Data 1 (p. 9, lines 223-227;

Extended Data legend, p. 29).

5 Comment 7: Figure 1D trace colors Comment: In Figure 1D, what do the blue and black traces mean, respectively? Response: We apologize that the trace identities were not explicit. Both are representative CA1 traces from the 8-week-old control condition. The black trace was recorded under normal ACSF(no GZ), and the blue trace was recorded after gabazine application (+GZ). The arrowhead associated with each trace marks its respective peak response (PEAK), as defined by the maximum value within the analysis window.

Changes in the manuscript: We revised the Figure 1D legend to identify the preparation, the black normal-ACSF trace, the blue gabazine trace, and the meaning of both arrowheads. We also clarified the arrowhead description in the PEAK definition in Materials and Methods (p. 8, lines 180-185; Figure 1D legend, p. 25, lines 649-651).

Comment 8: Figure 1F discrepancy Comment: In Figure 1F, the figure legend describes how the SUM maps were computed.

However, the figure itself shows how the average maps were obtained. Please correct the discrepancy.

Response: We thank the Reviewer for identifying this discrepancy. We revised the panel labels and legend so that the sequence from the slice-level response metric map to the across-slice average map is described correctly and consistently.

Changes in the manuscript: Figure 1F and the corresponding legend text were corrected (Figure 1F and legend, p. 25, lines 651-655).

Comment 9: Supplementary p-value color scale Comment: In Supplementary Figure 1C, the color scale of the p value is confusing. Please revise the color scheme to improve clarity.

Response: We agree that the original p-value color scale was difficult to interpret because lower p values were represented by darker colors that could blend with the grayscale anatomical background. We revised Extended Data Figure 2-1 using a perceptually ordered, color-vision accessible reversed viridis scale, in which lower p values are displayed with brighter colors. Pixels with p {greater than or equal to} 0.05 are left uncolored, and the color bar is now explicitly labeled "Uncorrected p" with numerical ticks at 0.001, 0.01, and 0.05. The same scale is used across all panels. We also clarified in the legend that these maps show uncorrected pixel-wise p values, whereas the BH-FDR-adjusted q-value maps are presented in Figures 2 and 3.

Changes in the manuscript: Extended Data Figure 2-1 and its legend were revised to use the same reversed viridis p-value scale across all panels and to state explicitly how uncorrected p values are displayed (Extended Data Figure 2-1 and legend, p. 30, lines 713-724).

6 We again thank the Editor and Reviewers for their constructive comments.
